# Long Non-coding RNA MRUL Contributes to Osteosarcoma Progression Through the miR-125a-5p/FUT4 Axis

**DOI:** 10.3389/fgene.2020.00672

**Published:** 2020-06-25

**Authors:** Cong Tian, Xingxing Sun, Kun Han, Hongling Zhu, Daliu Min, Shuchen Lin

**Affiliations:** ^1^Department of Medical Oncology, Shanghai University of Medicine & Health Sciences Affiliated Sixth People’s Hospital East Campus, Shanghai, China; ^2^Department of Medical Oncology, Shanghai Jiao Tong University Affiliated Sixth People’s Hospital, Shanghai, China; ^3^Department of Medical Oncology, Fudan University Shanghai Cancer Center, Shanghai, China

**Keywords:** osteosarcoma, long non-coding RNA, MRUL, miR-125a-5p, FUT4

## Abstract

Osteosarcoma (OS) originates in the skeletal system and has a rising global incidence. Long Non-coding RNAs (lncRNAs) are key regulators of human cancers development and progression. However, their roles in the development of OS are not well understood. This research aimed to investigate the effect of a long non-coding RNA (lncRNA), MRUL, on OS and revealed its potential molecular mechanisms. The bioinformatics analysis demonstrated that lncRNA MRUL was involved in regulating nucleic acid-templated transcription, cellular macromolecule biosynthetic process, immune response, and inflammatory response. In this work, the expression of lncRNA MRUL was detected by quantitative real-time polymerase chain reaction (qRT-RCR) in both cancer tissues and cell lines. We found that lncRNA MRUL was up-regulated in cancer tissues and cell lines. Functional experiments showed that knockdown of lncRNA MRUL inhibited OS cell proliferation, and metastasis. At the same time, we found that lncRNA MRUL interacted with miR-125a-5p to suppress FUT4 expression. Moreover, inhibition of miR-125a-5p abrogated the biological roles of lncRNA MRUL knockdown on OS cell proliferation, migration, and invasion. In conclusion, these results demonstrated that OS-upregulated lncRNA MRUL promoted cell proliferation, and metastasis via negatively regulating miR-125a-5p, and imply that lncRNA MRUL may be a potential biomarker for OS.

## Introduction

Long non-coding RNAs are linear RNAs that contain over 200 nucleotides in length (Chen et al., 2014). It accounts for about 95% of the total RNA, is widely distributed in the nucleus and cytoplasm and transcribed in eukaryotic cells (Gromesová et al., 2016). LncRNA played important roles in regulating gene expression in cells (Beermann et al., 2016; Fabbri et al., 2019). It was found that there were significant differences between tumor and normal tissues, indicating that lncRNA is important in the progression of human tumors (Wang et al., 2016). In prostate cancer cells, for example, knockdown of RP11-191L9.4 expression suppressed cell proliferation, migration and invasion ability (Yang and Song, 2019). According to the research of Loewen et al. (2014), lncRNA HOTAIR is highly expressed in lung cancer, associates with metastasis and poor prognosis. HOTAIR can also promote lung cancer cell proliferation, and drug resistance. Moreover, in gastric cancer cells, the role of H19 is mediated by direct up-regulation of ISM1 as well as the indirect inhibition of CALN1 expression (Li et al., 2014). According to the research of Wang et al., in gastric cancer, Bioinformatics analysis demonstrated that MRUL might positively affect ABCB1 expression in an orientation- and position-independent manner (Wang et al., 2014).

MicroRNA (miRNA) is a class of single-strand non-coding RNAs (Shikeeva et al., 2016). MicroRNAs bind to specific sites of messenger RNAs (mRNAs) to negatively regulate gene expression (Rico-Rosillo et al., 2014). In cells, lncRNAs and miRNAs can interact with each other and participate in regulating the metabolic activities of cells. For example, lncRNAs can act as miRNA sponges, reducing their suppression of mRNAs. TP53 is an important tumor suppressor gene (Paraskevopoulou and Hatzigeorgiou, 2016), which is closely related to the occurrence of human cancers. Previous studies demonstrated that miRNA, mRNA and lncRNA may participate in the downstream signaling pathway of p53, playing an important part in the carcinogenesis (Gong et al., 2015).

The incidence of OS is much lower than that of many other cancers, however, it is a malignant tumor (Ottaviani and Jaffe, 2009). OS tends to occur in adolescents in the distal femur or proximal tibia (Meyer, 1991). There are many unanswered questions about this cancer, including the exact cause of OS. In recent years, a number of researches on molecular mechanisms had contributed to the pathogenesis of OS (Gorlick and Khanna, 2010). Studies have shown that, compared with the normal tissues, lncRNA HULC is upregulated in tissues and cell lines of OS and associated with clinical staging and distant metastasis. In addition, a higher expression level of lncRNA HULC corresponds to a shorter survival of patients with OS. On the contrary, the decreased expression of lncRNA HULC can inhibit the development of OS cells (Sun et al., 2015). It is found that lncRNA-UCA1 was highly expressed in tissues of OS, which has a significant correlation with high tumor grade, large tumor growth and positive distant metastasis. *In vitro* experiments in cell lines of OS, lncRNA UCA1 knockdown inhibited cell proliferation, invasion and migration, and accelerated cell apoptosis, while lncRNA-UCA1 overexpression had the opposite effects (Braicu et al., 2013).

A previous study in gastric cancer showed long non-coding RNA MRUL promoted ABCB1 expression in multidrug-resistant cell sublines. However, the roles of this lncRNA in OS remained largely unknown. In this study, we demonstrated that lncRNA MRUL was highly expressed in OS cells. *In vitro* experiments, for OS cells, the downregulation of lncRNA MRUL inhibited the proliferation and weakened the migration and invasion ability. This suggests that lncRNA MRUL could be involved in the occurrence and progression of OS and could be a potential target for the treatment of this disease.

## Materials and Methods

### Bioinformatics Analysis

The DAVID^1^ tool was applied to analyze the enriched GO terms of lncRNA MRUL co-expressing genes based on the hypergeometric distribution to compute values. FDR < 0.05 was set as the threshold value.

### Integration of Protein-Protein Interaction (PPI) Network

STRING database (version 10.5) was used to evaluated the PPI information of LncRNA MRUL co-expressing genes w. Cytoscape software (version 3.6.1) was used to constructed the PPI networks.

### Cell Lines and Cell Culture

The normal human bone cell line hFOB 1.19, the OS cell lines SW1353, U2OS, and MG-63 were from ATCC. We cultured cells in DMEM (BI, Israel), supplemented them with 10% FBS (BI, Israel) and maintained them in a humidified incubator at 37°C with 5% CO_2_.

### Small Interfering RNA and Transfection

MRUL siRNA and Negative siRNA were synthesized by the Shanghai GenePharma Co. OS cells were transfected with siRNAs by Lipofectamine 2000 (Invitrogen, Calif.). MRUL siRNA-1 5′–GGCCUUUGUUUGCAGUUUATT–3′; MRUL siRNA-2: 5′–UUUCUACUGUUACUGUGUCTT–3′; Negative siRNA: 5′–UUCUCCGAACGUGUCACGUTT–3′.

### Cell Fractionation Assay

We follow the cell fractionation assay according to the previous report (Yi et al., 2018).

### Dual-Luciferase Reporter Assay

In order to build luciferase reporter vectors, the whole lncRNA MRUL or the 3′UTR fragment of FUT4 that contains the expected latent binding sites, were cloned into the pmiR-RB-REPORT^TM^ luciferase reporter vector at the *Xho*I and *Not*I sites. The mutant sequence of lncRNA MRUL or FUT4 at the 3′UTR was also built into the vector.

As for luciferase activity assay, we applied Lipofectamine 2000 (Invitrogen) to co-transfect each construct with marked miRNAs (RiboBio) in OS cells for 48h. They were performed with the Dual-Luciferase Reporter Assay System with the instruction of the manufacturer. Moreover, we quantified the Luminescent signals by BioTek Synergy HTX multi-mode reader, and presented the luciferase activities by relative hRluc/hluc ratio.

### qRT-PCR

We extracted total RNA from tissues and cells with TRIzol reagent (TAKARA, Dalian, China). For lncRNA and mRNA, the reverse transcription of total RNA to cDNA was performed by Reverse Transcriptase (Vazyme, Nanjing, China). Then we conducted qPCR with a SYBR Green PCR Kit (Vazyme, Nanjing, China). The fluorescence quantitative PCR instrument was QuantStudio^TM^ 6 Flex manufactured by Thermo Fisher Scientific (United States). Furthermore, Sangon (Shanghai, China) was used to construct all the primer sequences. MRUL(F): 5′–ACCCACAGACAACTGTGGACCC–3′, MRUL(R): 5′–GCCGCCCCTATTGTTGCCCA–3′; GAPDH (F): 5′–ACCCAGAAGACTGTGGAGG–3′, GAPDH (R): 5′–TTCTAGACGGCAGGTCAGGT–3′. The reference gene for lncRNA and mRNA was chosen as GAPDH. The internal control for miRNA was set as U6. We also applied the 2^–Δ^ ^Δ^ ^Ct^ method to quantify the gene expression.

### Cell Counting Kit-8 Proliferation Assay

We assessed OS cells proliferative ability with the CCK-8 assay according to the manufacturer’s instruction. Next, we plated SW1353 and U2OS cells (1 × 10^3^) in 96-well plates and treated them with 10 μl CCK-8 solution for 2 h, and then analyzed the spectrophotometrically at 450 nM by automatic microplate reader (HEALES Shenzhen, China).

### Transwell Migration and Invasion Assays

Transwell chamber was used to conduct the assays of cell migration and invasion, which was coated with the matrigel mix for invasion assay or without it for migration assay. After incubation for 48 h, we use cotton swabs to scrape the cells which were settled on the upper surfaces of the transwell chambers, and to fix the cells settled on the lower surfaces. Next, we observed the number of cells in the transwell chambers under a fluorescent inverted microscope and took a photo.

### Statistical Analysis

We applied the average value ± SD to express continuous variables and performed one-way ANOVA or Student’s *t*-test for multiple comparisons. In this paper, a statistically significant difference was demonstrated by *p* = 0.05.

## Results

### Co-expression Analysis of lncRNA MRUL in OS Cells

In order to investigate the molecular functions of lncRNA MRUL in OS, we extracted MRUL co-expressing genes in OS using cBioportal database^2^. The top 1000 positively and negatively correlated genes were selected as the potential targets of lncRNA MRUL in OS (Supplementary Table 1).

**FIGURE 1:**
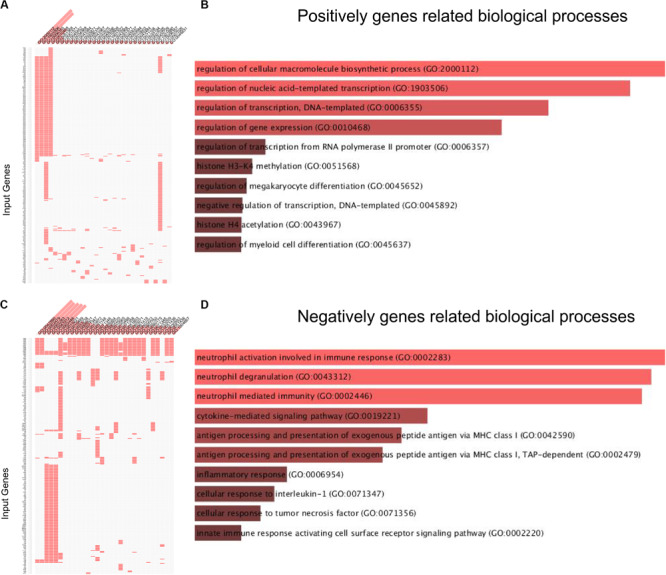
Biological processes analysis related to lncRNA MRUL in OS. **(A,C)** Positively/Negatively genes enriched in key GO terms. The abscissa represents GO term ID; the ordinate represents genes ID. **(A)** Positively genes; **(C)** negatively genes. **(B,D)** Bar-plot of biological processes analysis. The length of bar represents the gene counts; the color depth of bar represents the significance of enrichment **(B)** Positively genes; **(D)** negatively genes.

Then, we used the DAVID system to conducted bioinformatics analysis of lncRNA MRUL using its positively and negatively correlated genes. The results showed that lncRNA MRUL positively co-expressing genes were associated with the regulation of cellular macromolecule biosynthetic process (GO:2000112), nucleic acid-templated transcription (GO:1903506), transcription (GO:0006355), gene expression (GO:0010468), histone H3-K4 methylation (GO:0051568) (Figures 1A,B). And lncRNA MRUL negatively co-expressing genes were associated with the neutrophil activation involved in immune response (GO:0002283), cytokine-mediated signaling pathway (GO:0019221), antigen processing and presentation of exogenous peptide antigen via MHC class I (GO:0042590), inflammatory response (GO:0006954) (Figures 1C,D).

**FIGURE 2:**
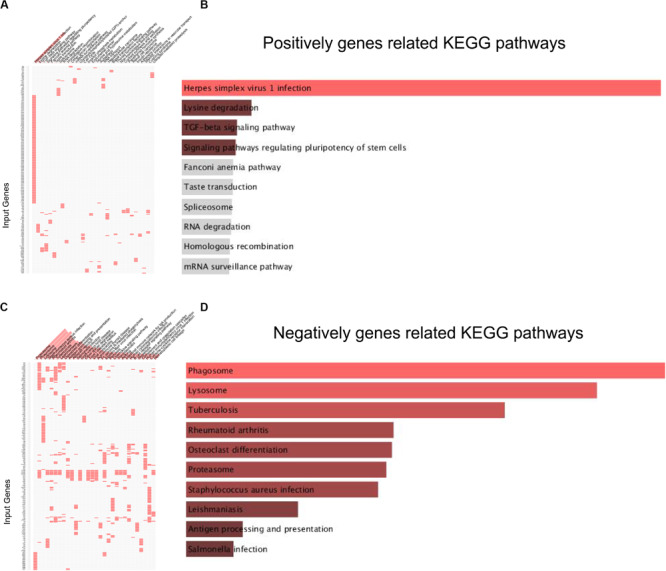
KEGG analysis related to lncRNA MRUL in OS. **(A,C)** Positively/Negatively genes enriched in key KEGG terms. The abscissa represents KEGG term ID; the ordinate represents genes ID. **(A)** Positively genes; **(C)** negatively genes. **(B,D)** Bar-plot of KEGG analysis. The length of bar represents the gene counts; the color depth of bar represents the significance of enrichment **(B)** Positively genes; **(D)** negatively genes.

KEGG pathway analysis revealed that lncRNA MRUL positively co-expressing genes were involved in regulating Lysine degradation, TGF-β signaling pathway, signaling pathways regulating pluripotency of stem cells, Fanconi anemia pathway, Taste transduction, Spliceosome, RNA degradation, Homologous recombination, mRNA surveillance pathway, other glycan degradation, and Cell cycle (Figures 2A,B). LncRNA MRUL negatively co-expressing genes were involved in regulating Phagosome, Lysosome, Tuberculosis, Rheumatoid arthritis, Osteoclast differentiation, Proteasome, Staphylococcus aureus infection, Leishmaniasis, and Antigen processing and presentation (Figures 2C,D).

### Construction of lncRNA MRUL Related PPI Networks in OS Cells

**FIGURE 3:**
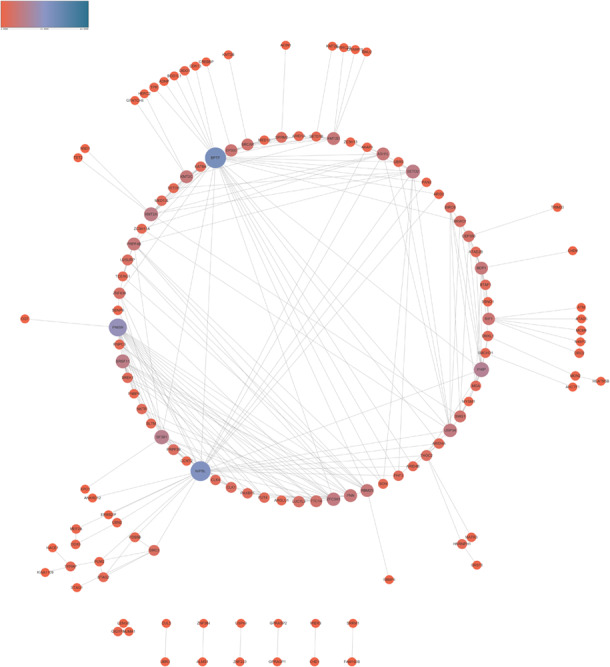
Co-expression network analysis positively related to lncRNA MRUL in OS. The size and color of node represents the degree. Connectivity degree is positively related to the size and color depth of nodes.

**FIGURE 4:**
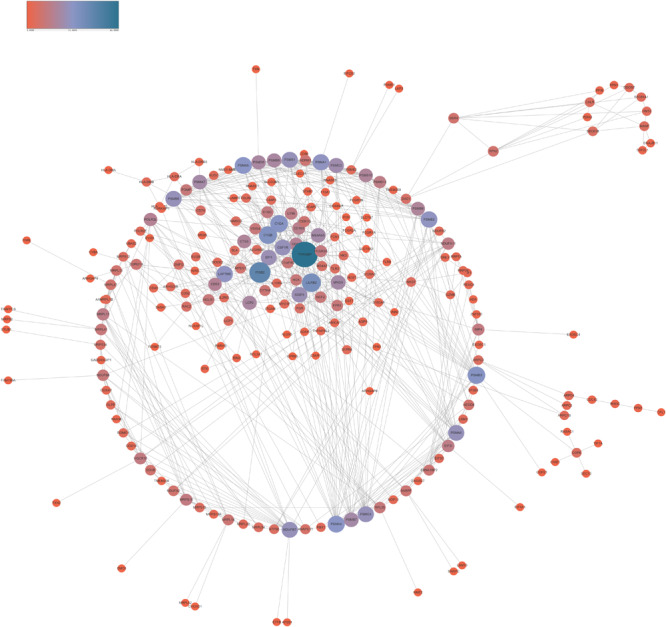
Co-expression network analysis negatively related to lncRNA MRUL in OS. The size and color of node represents the degree. Connectivity degree is positively related to the size and color depth of nodes.

In order to reveal the interaction among lncRNA MRUL positively and negatively correlated genes, we constructed PPI networks using STRING database. As present in Figure 3, the positive PPI network included 597 genes and 3467 edges. The negative PPI network included 844 genes and 10417 edges (Figure 4). Among these genes, ITGAM, ITGB2, IL10, UBA52, LILRB2, B2M, HCK, CCL4, CYBB, BTK, CCL5, CD48, FPR1, LY86, CD86, PSMB3, CDC42, FPR2, MNDA, CASP1, CXCL1, VAV1, FGR, PTPN6, MRC1, TNFRSF1B, SYK, TLR8, AIF1, C1QA, FCGR3A, NCF4, PRF1, PSMA1, CD163, CTSS, FCGR1A, HLA-DRA, CCL3, TXN, FCGR2B, CD300LF, PSMC3, ALOX5AP, C1QC, CFL1, HLA-B, MS4A6A, WAS, SETD2, CHD1, and SIN3A were identified as hub genes of lncRNA MRUL related PPI networks.

### LncRNA MRUL Was Highly Expressed in OS Cells

**FIGURE 5:**
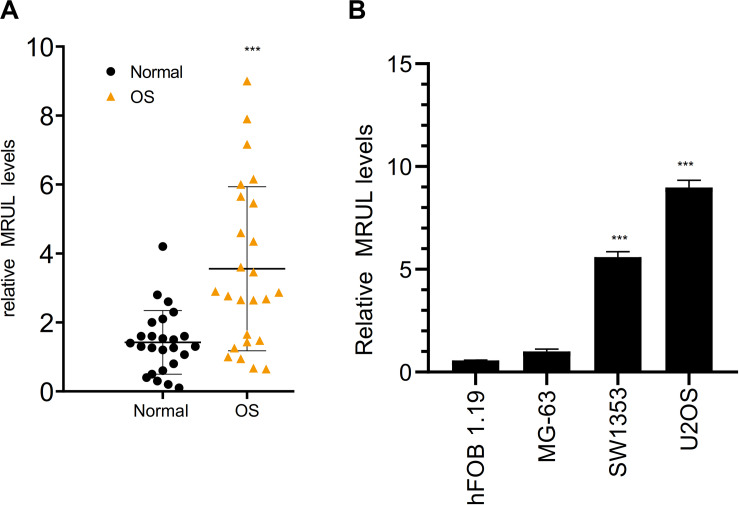
LncRNA MRUL was highly expressed in OS. **(A)** The expression of lncRNA MRUL in OS tissues was significantly higher than that in normal tissues. **(B)** By qRT-PCR, the expression of lncRNA MRUL in SW1353 and U2OS was five to eight times higher than that in normal cells (hFOB 1.19). Significance was defined as *p* < 0.05 (***p < 0.001).

**FIGURE 6:**
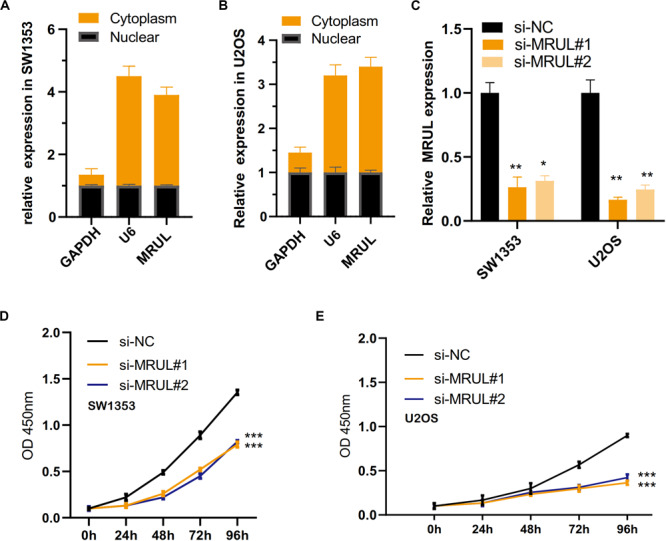
LncRNA MRUL was more located in the cytoplasm of the two OS cell lines and knockdown of lncRNA MRUL suppressed OS cells proliferation. **(A,B)** LncRNA MRUL was mainly located in the cytoplasm of the SW1353 **(A)** and U2OS **(B)** cells. **(A)** The efficiency of lncRNA MRUL knockdown was detected by qRT-PCR. **(D,E)** CCK-8 experiments showed that lncRNA MRUL knockdown attenuated the proliferative capacity of SW1353 **(D)** and U2OS **(E)** cells. Significance was defined as *p* < 0.05 (*p < 0.05; **p < 0.01; ***p < 0.001).

By qRT-PCR, lncRNA MRUL expression in OS tissues was significantly higher than in normal tissues (Figure 5A). Besides, lncRNA MRUL expression in different OS cell lines (MG-63, U2OS, and SW1353) and hFOB 1.19 cells was explored, we found that the expression was upregulated in the OS cells compared to that in hFOB 1.19 cells (Figure 5B). Moreover, we revealed that lncRNA MRUL was more located in the cytoplasm of the SW1353 (Figure 6A) and U2OS (Figure 6B) cells, suggesting the progression of OS in the cytoplasm may be regulated by this lncRNA.

### Knockdown of lncRNA MRUL Suppressed OS Cells Proliferation

To explore the impact of lncRNA MRUL on the biological function of OS cells, we use siRNAs (siMRULs) (Figure 6C) to silence the expression of lncRNA MRUL in SW1353 and U2OS cells. CCK-8 assays were used to measure its effect. It showed that knockdown of lncRNA MRUL could attenuate the proliferation ability of the SW1353 and U2OS cells (Figures 6D,E).

### Knockdown of lncRNA MRUL Suppressed OS Cell Metastasis *in vitro*

**FIGURE 7:**
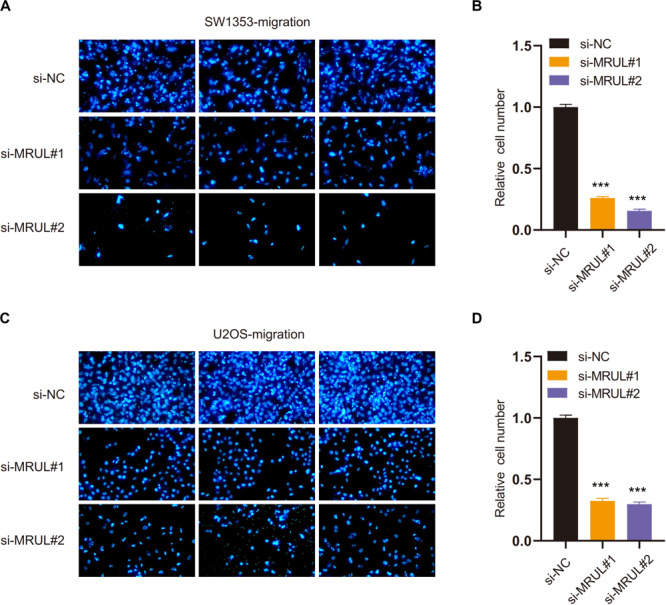
Knockdown of lncRNA MRUL suppressed OS cell migration. **(A,C)** The picture taken by a fluorescent inverted microscope showed that knockdown of lncRNA MRUL reduced cell migration ability [SW1353 **(A)**, U2OS **(C)**]. **(B,D)** The relative number of lncRNA MRUL knockdown cells was reduced by two-thirds compared to the control group [SW1353 **(B)**, U2OS **(D)**]. Significance was defined as *p* < 0.05 (***p < 0.001).

**FIGURE 8:**
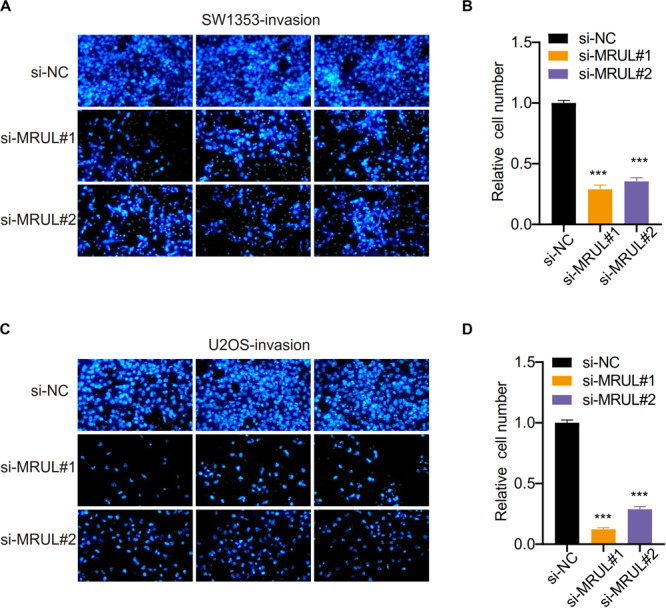
Knockdown of lncRNA MRUL reduced OS cell invasion. **(A,C)** Images of SW1353 **(A)** and U2OS **(C)** that can be counted after being treated with si-NC or si-MRUL or si-MRUL, respectively, under a fluorescence inverted microscope. The number of knockdown lncRNA MRUL cells was significantly less than the control group. **(B,D)** Compared with the control group, the number of lncRNA MRUL knockdown cells [SW1353 **(B)**, U2OS **(D)**] was reduced by more than half. Significance was defined as *p* < 0.05 (***p < 0.001).

We had constructed lncRNA MRUL knockdown cell lines by transfecting cells with the siMRULs and detected the efficiency by the transwell experiments. It could be seen that the migration and invasion capabilities of these lncRNA MRUL knockdown SW1353 (Figures 7A,B, 8A,B) and U2OS (Figures 7C,D, 8C,D) cells were significantly inhibited.

### LncRNA MRUL Promoted OS Cell Progression by Regulating miR-125a-5p/FUT4 Axis

**FIGURE 9:**
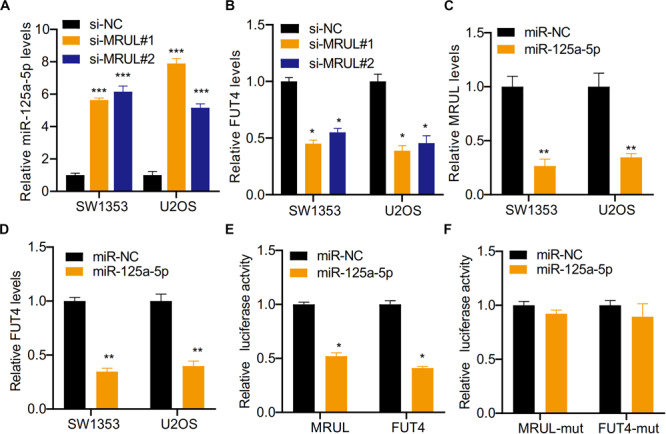
LncRNA MRUL promoted OS cell progression by regulating miR-125a-5p/FUT4 axis. **(A)** The relative miR-125a-5p levels in cells transfected with siMRULs increased more than fivefold compared to transfected with si-NC. **(B)** The expression levels of FUT4 in OS cells transfected with siMRULs were significantly reduced. **(C)** The relative MRUL levels of cells transfected with miR-125a-5p mimics were approximately one-third that of the control group. **(D)** The expression levels of FUT4 in OS cells transfected with miR-125a-5p mimics were significantly reduced. **(E)** Luciferase activity in OS cells co-transfected with lncRNA MRUL or FUT4 and miR-125a-5p mimics or miR-NC. **(F)** The luciferase activities of lncRNA MRUL-mut and FUT4-mut were measured OS cells transfected miR-125a-5p mimics or miR-NC with dual luciferase reporter assay. Significance was defined as *p* < 0.05 (*p < 0.05; **p < 0.01; ***p < 0.001).

It was found that the expression of target genes was regulated by a growing number of lncRNA via sponging miRNA. The bioinformatics analysis showed that miR-125a-5p could be sponged by lncRNA MRUL. Besides, lncRNA MRUL knockdown was found to significantly upregulate the expression of miR-125a-5p and down-regulate FUT4 in SW1353 and U2OS cells with qRT-PCR (Figures 9A,B). On the other side, it was shown that overexpression of miR-125a-5p declined lncRNA MRUL and FUT4 expression levels in SW1353 andU2OS cells (Figures 9C,D). Additionally, we constructed lncRNA MRUL, lncRNA MRUL-mut, FUT4, and FUT4-mut, co-transfected each of them into cells with miR-NC or miR-125a-5p mimics and performed luciferase reporter assay. It turned out that miR-125a-5p mimics inhibited the luciferase activity of lncRNA MRUL and FUT4 significantly (Figure 9E), but not lncRNA MRUL-mut or FUT4-mut plasmids (Figure 9F).

## Discussion

This present study explored the potential functions of lncRNA MRUL in OS using bioinformatics analysis and functional validation. The bioinformatics analysis demonstrated that lncRNA MRUL was involved in regulating cellular macromolecule biosynthetic process, nucleic acid-templated transcription, immune response, and inflammatory response. Through qRT-PCR, the expression of lncRNA MRUL in cancer tissues was found to be significantly more than that in normal tissues. *In vitro* experiments, siMRULs were used to silence its expression in SW1353 and U2OS cell lines. The results indicated that lncRNA MRUL knockdown reduced the proliferation ability of SW1353 and U2OS cells, and inhibited the migration and invasion ability of OS cells *in vitro*. To investigate the regulatory mechanism of lncRNA MRUL in OS, we found that lncRNA MRUL promoted the progress of OS cells by regulating the miR-125a-5p/FUT4 axis.

In recent studies, it is shown that lncRNAs are involved in multiple processes in cancer progression. They can interact with DNA, RNA and protein molecules, as well as complexes of DNA, RNA and proteins to enhance or inhibit the functions of these molecules and play a significant role in gene expression, which enhances the ability of cancer cells to differentiate, grow and metastasize (Yang et al., 2014; Han et al., 2015). LncRNAs can act as miRNA sponges or act directly on miRNA. For instance, HULC in liver cancer acted as an endogenous “sponge” to downregulate the activity of a range of miRNAs (Xin et al., 2018); microRNA-125a affects the occurrence and development of human liver cancer cells (Potenza et al., 2017b); MicroRNA−125a−5p is a downstream effector of sorafenib in its antiproliferative activity toward human hepatocellular carcinoma cells (Potenza et al., 2017a). Study in metastatic gastric cancer cells has found that miR-223, under the induction of Twist, down-regulates the expression of the key target gene EPB41L3 by directly targeting its 3′-non-translation region, enhancing the metastasis of cancer cells (Li et al., 2011). Moreover, the binding of RNA-binding protein HuR to lincRNA-p21 facilitates the recruitment of let-7/Ago2 to lincRNA-p21, resulting in decreased lincRNA-p21 stability (Yoon et al., 2012).

There is growing evidence that dysregulation of microRNAs (miRNAs) are involved in the progression of cancer. By analyzing the ectopic expression and target sites and of HCC cells, it was found that miR-125b and miR-125a-5p inhibited the expression of cyclin D1 and SIRT7, but induced p21WAF1/Cip1−dependent G1 cell cycle arrest (Kim et al., 2013). Sirtuins play a role in cell metabolism, anti-stress, and senescence, and the role of sirtuin7 (SIRT7) in ribosomal gene transcription has been proposed (Tsai et al., 2012). The downregulation of SIRT7 affects the cell cycle, leading to a substantial increase in cancer cells in G1/S phase, inhibiting growth. By detecting human lung cancer samples, the expression of miR-125a-5p in about one-third of the samples was found to be significantly down-regulated (Wang et al., 2009). Further studies on the cellular function of miR-125a-5p showed that it could negatively regulate the metastasis of cancer cells (Zhong et al., 2017). In addition, studies have shown that hsa-miR-125a-5p can induce human lung cancer cell apoptosis through a p53-dependent pathway. In addition, overexpression of hsa-miR-125a-5p can increase the protein and mRNA expression of wild-type p53, while blocking of wild-type p53 can weaken the impact of hsa-miR-125a-5p on apoptosis, and blocking of hsa-miR-125a-5p can reduce the mRNA and protein expression of wild-type p53 (Jiang et al., 2011). In OS, several studies demonstrated that miR-125a-5p acted as a tumor suppressor. For example, Niyazi et al. demonstrated that the downregulation of miR-125a-5p functions as a tumor suppressor by directly targeting MMP-11 in OS. Cao et al. showed miR-125a-5p was involved in regulating OS metastasis and mediated the effects of lncRNA HOXA11-AS on this progression. In this study, we found that the down-regulation of lncRNA MRUL significantly up-regulated the expression of FUT4 and miR-125a-5p in SW1353 and U2OS cells. Meanwhile, it showed that overexpression of miR-125a-5p reduced the expression levels of lncRNA MRUL and FUT4 in SW1353 and U2OS cells by qRT-PCR. Luciferase reporting experiments showed that miR-125a-5p inhibited luciferase activity of lncRNA MRUL and FUT4 significantly, while lncMRUL-mut or FUT4-mut were not affected by overexpressing miR-125a-5p.

Several limitations also existed in this study. First, despite the expression levels of lncRNA MRUL were detected in a small sample size OS samples, the correlation between the expression of MRUL, miR-125a-5p, and FUT4 and clinical parameters should be further validated using a bigger sample size, which could be helpful for us to understand the functional importance of this axis. Second, the findings of this study were concluded with *in vitro* assays. Therefore, the *in vivo* assays should also be conducted to further validate our findings.

## Conclusion

In conclusion, we demonstrated that the downregulation of lncRNA MRUL significantly up-regulated the expression of miR-125a-5p but suppressed the corresponding target gene FUT4, and inhibited the occurrence and invasion of OS. The results showed that lncRNA MRUL could be a promising target to treat the disease of OS.

## Data Availability Statement

All datasets generated for this study are included in the article/Supplementary Material.

## Author Contributions

SL and DM designed the research. CT and XS performed the research and wrote the manuscript. KH and HZ helped in the analysis of the data. All authors contributed to the article and approved the submitted version.

## Conflict of Interest

The authors declare that the research was conducted in the absence of any commercial or financial relationships that could be construed as a potential conflict of interest.

## Footnotes

1 http://david.abcc.ncifcrf.gov/

2 https://www.cbioportal.org/datasets
